# Atomistic Structure Investigation of Eu-Doped ZnO Nanosponges

**DOI:** 10.1021/acs.inorgchem.4c04494

**Published:** 2025-01-02

**Authors:** Shihui Feng, Sarmad Naim Katea, Markus Ek, Gunnar Westin, Cheuk-Wai Tai

**Affiliations:** † Department of Material and Environmental Chemistry, Arrhenius Laboratory, Stockholm University, SE-10691 Stockholm, Sweden; ‡ Department of Chemistry-Ångström, Ångström Laboratory, Uppsala University, SE-75121 Uppsala, Sweden

## Abstract

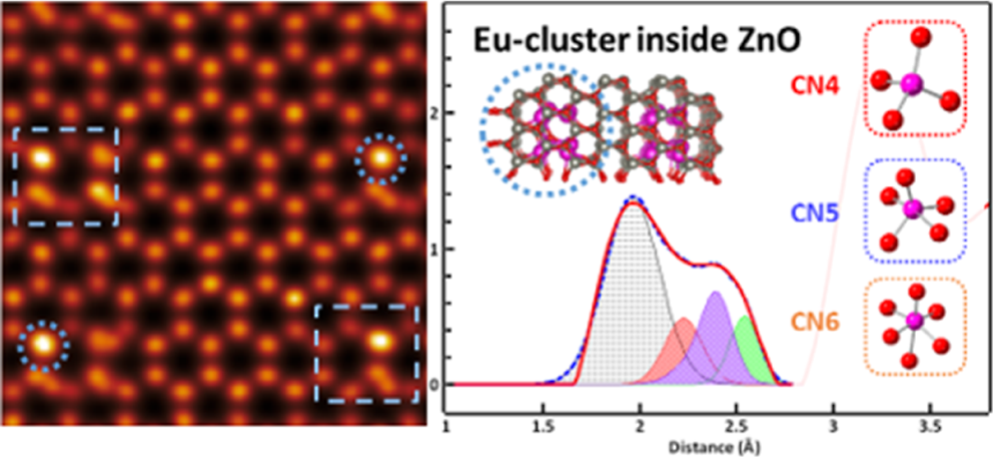

Zinc oxide (ZnO) is a semiconductor with a wide range of applications, and often the properties are modified by metal-ion doping. The distribution of dopant atoms within the ZnO crystal strongly affects the optical and magnetic properties, making it crucial to comprehend the structure down to the atomic level. Our study reveals the dopant structure and its contents in Eu-doped ZnO nanosponges with up to 20% Eu–O clusters. Eu was distributed over the ZnO:Eu crystals, with an additional amorphous intercrystalline phase observed, especially in the 20% Eu sample. The electron pair distribution function revealed the presence of nonperiodic Eu^3+^-oxide clusters and proved highly effective for analyzing the coordination environment of Eu–O, ranging from 2.0 to 2.8 Å. It uncovered three-, four-, and five-coordinate Eu–O configurations in the 20% Eu sample, and there were significant changes in Eu coordination between the samples, which is ascribed due to the intercrystalline phase. The proposed method offers a potential characterization routine for a detailed investigation of complex doped materials.

## Abstract 2

The Eu-cluster packing configuration within ZnO was successfully revealed. The distribution and existence of Eu elements were revealed by HR-STEM imaging, while the electron pair distribution function results indicate direct atom–atom bonding configurations and coordination environments. Our proposed method offers a promising characterization approach for detailed investigations of complex doped materials.

## 1 Introduction

Zinc oxide (ZnO) is a widely used abundant material of low toxicity showing a unique combination of properties including a wide band gap (3.4 eV), high electron mobility, wide range of optical transparencies, and room-temperature luminescence.^1−3^ These properties have led to many current applications and promises of application in diverse fields such as transparent electrodes,^4^ batteries,^5^ catalysis, photo-/electro-catalysis for water and gas remediation,^6−8^ solar-cells,^9−13^ and sensors.^14,15^ Doping with different ions, in particular d- and f-block elements, can be used to modify the structural, optical, electrical, and magnetic properties. Therefore, much attention is paid to metal doping of ZnO, especially when considering utilization of optic and magnetic properties in multifunctional devices.^16,17^ Many metal-ion dopants have sizes and coordination requirements, making them unlikely to fit the ZnO host structure, but nevertheless, there are many reports of high doping levels with these ions. One of the most studied group of dopants is that of the large, alio-valent Ln-ions^18^ where there has been an ongoing debate on the viability and nature of the claims of doping levels of 5 at. % Ln and higher. Another problem arising when trying to understand the effect of adding dopant ions is the often highly varying properties found with similar doping levels and even rather similar synthesis routes. These differences are likely the results of impurities, crystal quality differences, ion valence, and local dopant coordination. This is a general problem that has hampered progress in many important research fields such as solar cells, solar-hydrogen catalysis, and diluted magnetic semiconductors.^19,20^

Many applications of ZnO depend on good electronic transport while retaining a large available surface area interacting with electrolytes or species to be chemically converted or sensed in the gas or liquid phase. Nanocrystalline sponges combine intimate metal-oxide crystal interfaces while still possessing a large-surface area. Moreover, nanosponges may be produced from low-cost simple chemicals with extremely short heating times of a few minutes or less.^21^ Also, for the rational design of doped semiconductor oxides, it is of great interest to investigate the nature of the dopants within the ZnO structures. In previous work, the much-debated local dopant structure in Eu-doped ZnO nanosponges with as much as 5 mol % of EuO_1.5_ in ZnO was studied.^21,22^ It was found that europium oxide clusters form within the ZnO structure without much disturbance and change in the size of the ZnO lattice.

Therefore, the possibility of increasing the Eu-oxide doping level even beyond 5% was explored to find out how much of the Eu-oxide clusters can be accommodated within the ZnO structure. X-ray diffraction (XRD) and IR spectroscopy indicated a change in doping mode between 10 and 20 at. % Eu. SEM, XRD, and IR spectroscopy, used here, are techniques describing the average structure but do not provide information on the local structural changes on a very fine nano- or atomic-scale required to understand these complex materials. At these high doping levels, it is not unlikely that more than one kind of Eu-doped ZnO is present. Therefore, advanced HR-TEM techniques were used to describe the nanocrystalline ZnO:Eu sponges doped with 5 and 20 atom % Eu at scales from entire sponge particles, via the ca. 10–20 nm sized ZnO:Eu crystals and grain boundaries to the atomic scale. HAADF-STEM imaging, which strongly relies on the sample thickness and atomic number (*Z*), was used together with electron diffraction to reveal the structure at the atomic scale. To gain a comprehensive understanding of the local atomic structure and correlations between europium, zinc, and oxygen atoms, as well as structural ordering and a possible secondary phase, electron pair distribution function (PDF) was used to reveal the atom–atom distances in a chosen area. PDF shows the probability of finding an atom at a certain distance *r* from a given atom. This function can be mathematically described as follows
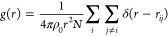
where *N* is the total atom number in our system studied, ρ_0_ is the average number density of atoms, *r*_*ij*_ is the distance between atom *i* and atom *j*, and δ(*r* – *r*_*ij*_) is the Dirac-delta function which equals a single unit only when *r* = *r*_*ij*_. The above equation illustrates the meaning of PDF, while each atom–atom distance could not be directly measured in a real experiment. The reduced PDF (rPDF) was commonly used to describe the structure features. This correlation function can be written as
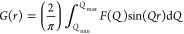
where *Q* is the momentum transfer vector or the so-called wave vector, *F*(*Q*) is the reduced structure function, and *Q*_max_ and *Q*_min_ are the maximum and minimum limitations of momentum transfer vector in the diffraction experiment, respectively. In addition to the atom–atom distance, the PDF also indicates structural features, including coordination number, average atom density, and atomic periodicity. During the last decades, PDF analysis was mainly based on X-ray and neutron scattering and became a well-established technique for the structural characterization of crystalline^23^ and amorphous materials.^24,25^ Here, by utilizing the advantages of the high spatial resolution and tunable illumination area given by TEM, the PDF was obtained in a TEM and the structure was investigated at nanoscale.

Five samples with different Eu doping concentrations 0, 5, 10, 20, and 33 mol % EuO_1.5_ in ZnO (given below as 0Eu, 5Eu, 10Eu, 20Eu, and 33Eu) heated at 600 and 650 °C for 3 min were investigated. From previous studies, it was found that 5Eu nanosponges could be obtained at 200 °C, 3 min. These sponges showed XRD patterns completely assignable to *h*-ZnO with very minor changes in unit cell parameters.^22^ However, TEM, XPS, TGA, and IR spectroscopy showed the presence of minor amounts of organic residues, which were replaced by Eu-oxo-carbonate when heating at 300–500 °C for 3 min. Heating at 600 °C for 3 min removed the carbonate and yielded a pure oxide 5Eu nanocrystalline sponge without detectable phase separation. Upon heating at 700 °C, TEM revealed a few emerging ca. 1–3 nm sized Eu_2_O_3_ crystals at the ZnO:Eu sponge surface which grew into nanosized *c*-Eu_2_O_3_ at higher temperatures. With lower Eu loading, the phase separation occurred at higher temperatures of up to 900 °C. The Eu-oxide was found to be homogeneously distributed within the ca. 10 nm sized ZnO:Eu crystals building up the nanosponges, while the XRD unit cell parameters were virtually unchanged. EXAFS and DFT modeling suggested that (EuO_1.5_)_4–8_ clusters were present inside the ZnO crystals, in excellent agreement with the experimental data.^22^ This surprising finding explained how large amount of Eu-ions can be incorporated into ZnO without much change of XRD unit cell parameters. With such high Eu-loading, there should only be less than ca. 2 nm of ZnO between the Eu-oxide clusters. The studies also indicated that the window of carbonate-free and nonphase-separated pure ZnO:Eu nanosponges extends to even higher Eu-doping levels.

It was therefore interesting to investigate in detail how high Eu-doping levels of the ZnO:Eu nanosponges can be accommodated without phase separation detectable with XRD and what structures form when exceeding the maximum Eu-doping limit. Herein, XRD and IR spectroscopy were used to determine the temperature limits for the presence of carbonate (lowest *T*) and phase separation (highest *T*) versus Eu-oxide concentration. Considering the doping model with 4 to 8 Eu-membered nonperiodic oxide clusters in crystalline *h*-ZnO given in literature,^22^ it should only be possible to reach 10–15% EuO_1.5_ (for distinguish, the chemical formula of EuO_1.5_ in this study indicates the europium–oxygen clusters inside ZnO nanocrystals) in the ZnO before there is not enough ZnO to cover each cluster with one layer of ZnO. However, XRD and IR spectroscopy describe the materials as an average over the volume of the material which does not allow for obtaining important details on where the Eu resides, its oxidation state, the Zn- and Eu-to-oxygen coordination spheres, and the presence of more than one phase. Therefore, an extensive study using TEM/STEM in real-space and reciprocal-space was used to provide the detailed structural features of the ZnO:Eu nanosponges. The STEM images and spectroscopy showed that Eu was not only present in the ZnO crystals but also formed an Eu(III)-oxide-rich intercrystalline amorphous phase, especially pronounced with the higher Eu-doping levels. The ePDF revealed atom–atom pair distances within the ZnO:Eu sponges, which further proved the existence of Eu-oxide within the ZnO as well as the Eu-rich intercrystalline boundary phase.

## 2 Experimental Section

### 2.1 Sample Preparation

#### 2.1.1 Nanosponges Synthesis

Zinc diacetate tetrahydrate [Zn(OAc)_2_·4H_2_O], zinc dinitrate hexahydrate [Zn(NO_3_)_2_·6H_2_O], triethanolamine (TEA), and methanol (p.a.) were used as received. The synthesis of precursor pastes with 0, 5, 10, 20, and 33 mol % EuO_1.5_ in ZnO was made in a similar way as previously described for the synthesis of ZnO:Eu pastes with up to 5 mol % EuO_1.5_. Shortly, zinc nitrate and acetate, respectively, were dissolved in methanol, and 0.3 TEA was added per Zn. Then, the zinc nitrate-TEA and zinc acetate-TEA solutions were mixed and evaporated until a clear or whitish paste was obtained. In the present study, the heat-treatment differed slightly from previous studies; instead of heating the paste directly at temperatures of 200 °C or higher for 3 min, it was first heated at 400 °C for 1 min and cooled to room-temperature, and thereafter, portions of it were heated at 600 or 650 °C, respectively, for 3 min. When microstructures and XRD patterns were compared, no significant difference was observed between the two techniques.

Safety: while we have not had any accidents with quite a number of syntheses made, any mixture of organics and nitrates should be regarded as potentially explosive, especially on heating and in large-scale synthesis.

#### 2.1.2 (S)TEM Sample Preparation

The powder samples were dispersed in ethanol and subjected to an ultrasonic treatment for 1 min. The dispersion was then dripped onto a lacey carbon copper grid. The grid was dried by using infrared light for 5 min. Then, the sample was placed in a plasma cleaner (Fischione Instruments model 1020) for 15 s to remove organic surface contamination.

### 2.2 Equipment Used

#### 2.2.1 X-ray Diffraction

Powder XRD patterns were obtained with a Bruker D8 equipped with a Lynxeye XE-T detector and using Cu Kα radiation (1.5460 Å) in the θ–2θ mode over the range 10–100° 2θ. Determination of unit cell-dimensions of the h-ZnO was achieved with Topas software version 6 applying the PDF card 00-036-1451 file from PDF-4+ as ZnO.

#### 2.2.2 IR

IR spectra were obtained in the mid- (4000–370 cm^–1^) and far-IR (1000–150 cm^–1^) ranges using a nitrogen-purged PerkinElmer spectrum, one instrument equipped with KBr and polyethylene beam splitters, for the mid- and far-IR ranges, respectively. The spectra were obtained with a resolution of 2 cm^–1^ for the mid-IR range and 4 cm^–1^ for the far-IR range. The powder samples were studied on a diamond-ATR (Pike GladiATR) unit.

#### 2.2.3 TEM

TEM data collection was performed using a double aberration-corrected Themis Z microscope (Thermo Fisher Scientific Inc.) equipped with a SuperX G2 EDX system. The aberration was corrected to fifth order. The experiments were carried out at a 300 kV accelerating voltage, including TEM, STEM, and spectroscopy work. The convergence angle of STEM high-angle annular dark-field imaging (STEM-HAADF) was set to 21 mrad with a probe current of 80 pA, while the inner and outer collection angles for the HAADF-STEM detector were selected as 64 and 200 mrad, respectively. To enhance the signal-to-noise ratio, the probe current was adjusted to 150 pA during the STEM energy dispersive X-ray (STEM–EDX) experiment. The EDX experiment was performed using a 1024 × 1024 scan size to maintain the spatial resolution by 4 μs dwelling time. The atomic fraction maps were selected to present the EDX mapping. For the chemical state analysis of europium and zinc, electron energy-loss spectroscopy (EELS) was applied. The EELS data were collected in the STEM mode using the Gatan Quantum GIF with a 29.5 mm camera length with 5 mm entrance aperture and a semicollection angle of 28.24 mrad. The low-loss and high-loss spectra were captured simultaneously in the dual-EELS mode. The SNR mode ([1130]*x* detector binning) was used for STEM-EELS to get high signal-to-noise ratio data. Exposure time for each pixel is 0.05 s with spatial drift correction every five rows. The electron diffraction patterns for the ePDF study were acquired in the TEM diffraction mode by using the Gatan OneView camera. To achieve a high q-range (resolution ring = 0.357 Å) while preserving the diffraction features, the camera length was set to 460 mm and the exposure time for each diffraction pattern was 2 s.

#### 2.2.4 Data Processing and PDF Simulation

The low-pass filter was applied to the HAADF-STEM images to remove the high-frequency noise. The electron-PDF can be calculated from the scattering profile. The Difftools,^26^ a Gatan DigitalMicrograph plugin package, was used to extract the scattering profile. SUePDF software^27^ was used for ePDF calculation, the average number density (ρ_0_) were obtained by calculate the slope between first physical peak and origin. Hence, the full PDF [*g*(*r*)] can be obtained by the following equation
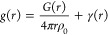
where γ(*r*) is the nanoparticle form factor dependent on the shape of the scatters. Considering the closely connected ZnO nanoparticles within our nanosponges, the form factor was simply set to 1 (bulk) in our case. Meanwhile, the coordination number (*N*_c_) can be calculated by the following equation
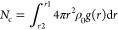


The STEM image simulation was carried out by Dr. Probe software.^28^ All image simulation parameters corresponded to the STEM data collection. The inner and outer collection angles of the HAADF-STEM detector were 64–200 mrad. The sample thickness was 8 nm, which equals the average particle size of ZnO nanosponges. The EELS data processing was carried out by DigitalMicrograph, including background subtraction and quantitative analysis of Zn and Eu. The PDF simulation was carried out by the Debye PDF method in the DiffPy-CMI package^29^ on the Eu8 oxide cluster structure.

## 3 Results and Discussion

### 3.1 Overall Phase Analysis of Eu-Doped ZnO

Overall phase analysis of Eu-doped ZnO with 0, 5, 10, 20, and 33 mol % EuO_1.5_ in ZnO (ZnO:0–33Eu) was made with powder-XRD and IR spectroscopy to investigate the metastable limit for Eu-oxide doping of ZnO without phase separation, i.e., with retained *h*-ZnO XRD patterns and Zn–O vibration peak in the IR spectra.

**Figure 1:**
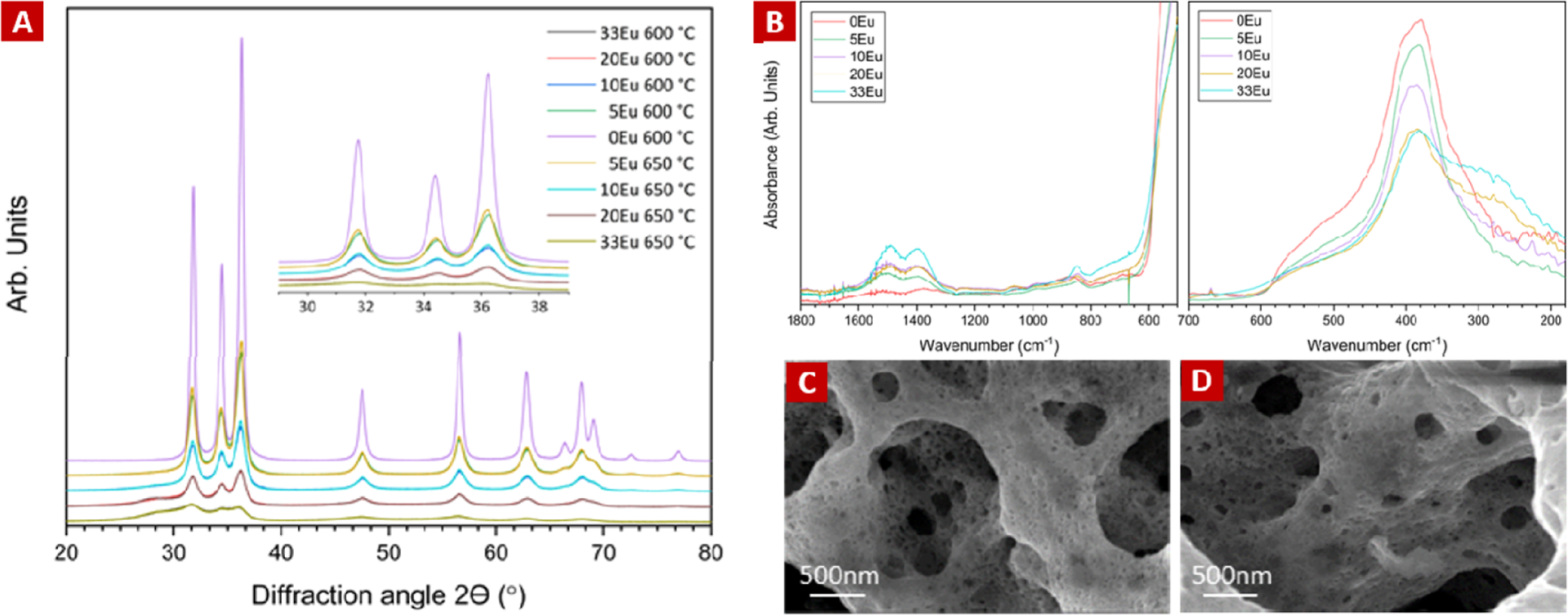
(A) XRD of 0, 5, 10, 20, and 33% EuO_1.5_ in ZnO heated at 600 and 650 °C for 3 min, respectively. The inset figure shows the diffraction at 28–40°. (B) IR spectra of ZnO:Eu nanosponges with 0, 5, 10, 20, and 33% EuO_1.5_ in ZnO heated to 650 °C (left) mid-IR range (1800–400 cm^–1^) and (right) far-IR range (700–180 cm^–1^). (C,D) SEM images of ZnO:Eu nanocrystalline sponges heated to 650 °C. (C) 5Eu and (D) 20Eu.

General structures of various Eu-doped ZnO sponges heated at 600 and 650 °C, respectively, are shown in Figure 1. The corresponding unit–cell parameters are given in Table S1. It can be observed that all sponges were quite similar to pure *h*-ZnO:Eu as the only identifiable crystalline phase after heating at different temperatures (600 and 650 °C). Very broad and overlapping peaks at ∼28.6 and ∼29.7° 2θ came up stronger while the ZnO:Eu peaks were strongly reduced in intensity with increased Eu-loading, which is associated with the nonperiodic [EuO_1.5_]_*n*_ clusters. The 33Eu sample showed very weak *h*-ZnO peaks overlapping the broad peaks, which prevented unit cell parameters from being extracted. The unit cell parameters of the Eu-doped *h*-ZnO:Eu phase were very similar for the two synthesis temperatures, differing in the *a*/*b*- and *c*-axis lengths only by <0.003 Å. The pure ZnO sample showed a larger difference in unit–cell parameters between the samples heated at 600 and 650 °C, which might be due to an easier relaxation of the lattice while removing defects when there are no Eu-oxide clusters present.

Infrared (IR) spectra were obtained for 0–33Eu nanosponges heated at 650 °C. The ZnO:0–20Eu nanosponges heated at 600 °C exhibited similar spectra to those heated at 650 °C, with minor differences. Notably, the 33Eu sample heated at 600 °C showed weak multiplet bands around 1060 and 1240 cm^–1^, absent in the 650 °C sample. All samples displayed weak peaks at 1580–1300 cm^–1^, attributed to symmetric and asymmetric C–O stretching in Eu-oxo-carbonate, along with water bending modes at 1640 cm^–1^. The Zn–O vibration peak at ∼390 cm^–1^ was stronger than the water and carbonate peaks at 840–860 cm^–1^. Additionally, a broad band at 350–200 cm^–1^, mainly associated with Eu–O vibrations in a noncrystalline state, intensified with higher Eu content. The Zn–O vibration band ranging from 600 to 300 cm^–1^ with a maximum at ca. 390 cm^–1^ was significantly reduced in intensity with increased Eu-content. In addition to the peak assigned to various Zn–O vibrations, a broad band was observed at 350–200 cm^–1^ which increased in relative intensity with Eu content, especially when comparing the 0–10Eu samples with the 20Eu and 33Eu samples. This band is assigned mainly to Eu–O vibrations in a noncrystalline state.

Thus, the XRD data indicated that as much as 33 mol % EuO_1.5_ may be present without formation of other crystalline phases such as *c*-Eu_2_O_3_. There was no strong trend in *h*-ZnO unit cell parameters among the very similar sized ZnO:Eu samples, although a very small reduction in unit cell-volume with increased Eu-content from 5 to 20% Eu was observed. The IR spectra gave similar results as the XRD patterns with the 0–10Eu samples showing similar Zn–O peaks and a very weak band associated with Eu–O vibrations, while the 20Eu and more so the 33Eu sample showed a relatively stronger Eu–O vibration band. The 33Eu sample also stood out with a differently shaped carbonate peak band, compared to the lower Eu-loadings. This indicates that at least 10% Eu can be present as Eu-oxide clusters in the ZnO crystal in a similar fashion as described for the previously studied 5Eu sample,^22^ while the 20Eu sample seems to have at least partially some other mode of Eu-doping, and the 33% Eu may have a substantial part of the Eu-oxide doping in a different way than found for the 5Eu sample. The SEM images presented in Figure 1C,D show overall ZnO:Eu sponge microstructures with a wide range of pore sizes from nm in the ZnO:Eu walls to μm between the walls. Thus, the structures were very similar for the 5Eu and 20Eu sponges. No signs of phase separation were observed on this scale.

### 3.2 Atomic-Scale Structure Characterization

The XRD and IR studies indicated that the 10Eu sample had a similar doping mode as the 5Eu sample, while the 20–33Eu sponges showed differences, indicating that a different doping mode was at least partially present. As mentioned above, from structural considerations, extending the (EuO_1.5_)_4–8_ oxide cluster doping model previously obtained with EXAFS, XRD, and DFT for the 5Eu samples^22^ seem possible only to ca. 10–15% Eu with the higher concentration for the larger oxide clusters with eight Eu-atoms. At higher doping levels, there would not be enough ZnO to surround the Eu-oxide clusters. Therefore, a detailed study was conducted on the 20Eu sample to find out how Eu is distributed in this sample. The 5Eu sample was studied for comparison after heating to 650 °C in similarity with the 20Eu sample.

**Figure 2:**
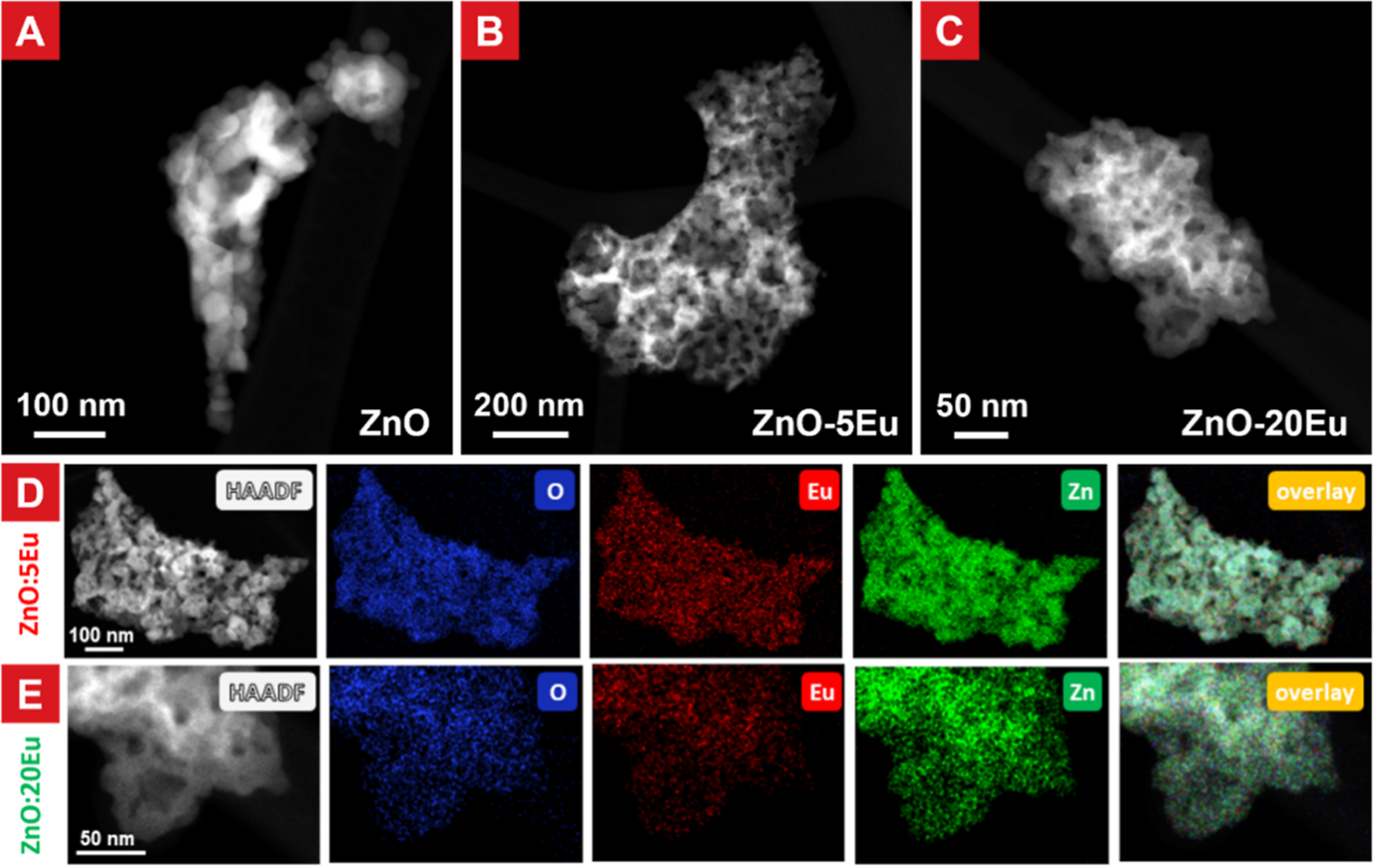
Morphology and EDX element mapping of ZnO:Eu nanosponges. (A–C) HAADF-STEM images of ZnO (A), 5Eu (B), and 20Eu (C) samples showing the nanosponge features. STEM–EDX mapping: 5Eu (D) and 20Eu (E) showing an even distribution of zinc, oxygen, and europium without obvious phase separation for both samples. Regional EDX spectra of doped samples, as well as EDX mapping of pure ZnO nanosponges are provided in the Supporting Information.

HAADF-STEM was used to distinguish europium containing phases, where the large difference in the atomic number for zinc (30) and europium (63) provides good contrast. Figure 2A–C shows the overall morphology of ZnO:Eu sponges with 0 (pure ZnO), 5 and 20Eu compositions. The ZnO:Eu sponge microstructure are similar to those observed by SEM displaying varied pore sizes and ZnO:Eu sponge walls consisting of closely connected nanocrystals in sizes ranging from 6 to 8 nm. A difference between doped and nondoped sponges observed by STEM was that the doped samples (5Eu and 20Eu) had a higher degree of nanoporosity within the ZnO:Eu crystal-walls, in comparison with the nondoped ZnO. STEM–EDX mapping of the 5Eu sample (Figure 2d) and 20Eu sample (Figure 2e) showed the presence and distribution of oxygen (O), zinc (Zn), and europium (Eu). The EDX spectra and element compositions are provided in the Supporting Information (Figures S1–S3).

**Figure 3:**
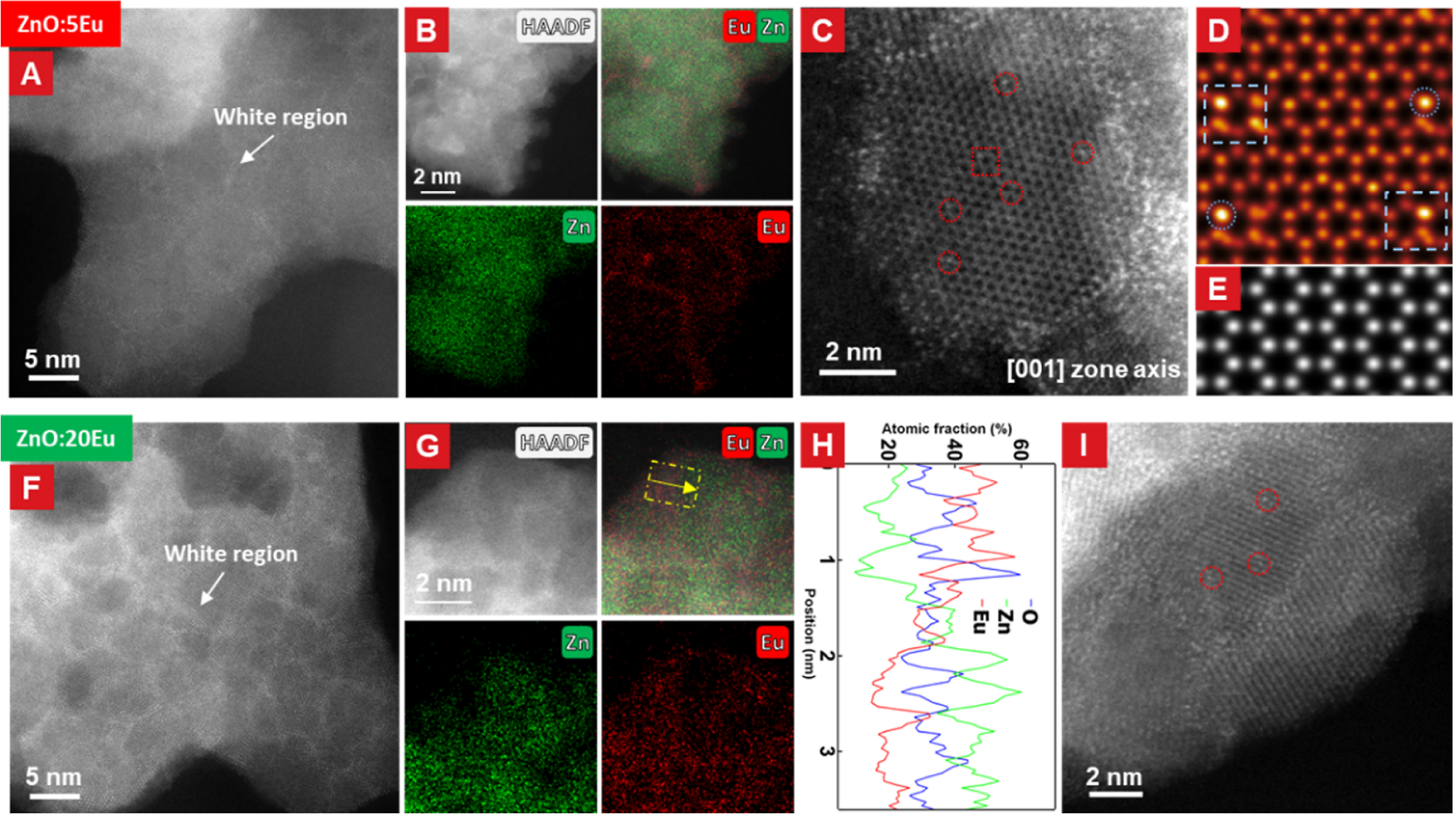
Structure characterization of ZnO:Eu samples. (A,F) HAADF-STEM images of 5Eu and 20Eu samples showing some bright-contrast regions in HAADF-STEM images indicating Eu-rich regions. (D,E) Simulated STEM-HAADF images of the DFT-calculated model with the [001] zone axis. (B,G) EDX showing an Eu-rich area between ZnO:Eu nanocrystals in the 5Eu and 20Eu samples. (H) STEM–EDX line profile extracted from (G), showing the Eu concentration difference between boundary and nanocrystal. (C,I) High-resolution HAADF-STEM of two samples. The square mark in C indicates the area which has lattice distortion caused by Eu atoms. The circle mark in (C,H) shows that the atom column contains Eu atoms with higher intensity.

The detailed structural characterizations of the ZnO:Eu nanosponges are presented in Figure 3. The HAADF-STEM images taken at higher magnification showed brighter contrast between the ZnO:Eu nanocrystals in both samples, suggesting an Eu-richer region. The 20Eu sample exhibited more of the Eu-rich intergranular regions, which were also wider at typically 5–10 Å thick, compared to the 5Eu sample. STEM–EDX mapping (Figure 3B,G) further confirmed the increase in Eu concentration in these intercrystalline regions. The EDX line profile of the highlighted region in the overlay in Figure 3G (Figure 3H) reveals the elemental concentration variation from the boundary toward the ZnO:Eu nanocrystal. It shows a discernible decrease in the Eu signal, accompanied by an increase in the Zn signal for the ZnO:Eu crystal, while the O concentration remains relatively stable. This shows a higher concentration of Eu presented at the grain boundaries of the ZnO:Eu nanocrystals. In the case of the 20Eu nanosponge, the extracted spectrum from two distinct local regions and subsequent calculation of the normalized composition yielded an approximate Eu concentration at the crystal boundaries of 13 atom % as compared to 3 atom % within the nanocrystals (Figure S4). A similar comparison for the 5Eu sample yielded results analogous to those obtained for the 20Eu sample (Figure S5) with the Eu concentration at the crystal boundaries and within ZnO:Eu crystal being approximately 6 at. % Eu and 2 at. % Eu, respectively.

Given the identification of Eu within the ZnO crystals and the observed segregation of Eu-oxide to the crystal boundaries, the distribution of Eu atoms within ZnO nanocrystals was investigated by atomic-resolution HAADF-STEM images (Figure 3C for 5Eu and Figure 3I for 20Eu). Some atomic columns with brighter contrasts indicated the presence of Eu within the ZnO structure. The simulated HAADF-STEM images were made by leveraging the doped structure^22^ and the hexagonal ZnO structure along the [001] zone axis. Certain brighter atomic columns in experimental data are marked by red circles in Figure 3C,I. Comparisons between the experimental data and simulated results, as well as our previous study, demonstrate a good fit for Eu atoms in ZnO nanocrystals. This revealed that Eu doping in the ZnO structure lacks long-range periodicity and exhibits a more random distribution. Moreover, it was found that the insertion of Eu atoms or clusters did not introduce significant lattice distortion into the ZnO lattice, which is in line with the XRD results. The Eu cluster is labeled by a square mark in both the experimental and simulation data (Figure 3C,D). To ensure the representativeness of these findings, additional data were collected that are presented in the Supporting Information (Figures S6 and S7).

Thus, real space investigation confirmed the presence of Eu atoms within the ZnO nanocrystals. The Eu-rich amorphous intercrystalline phase revealed was most significant for the 20Eu sample but was also present to some extent in the 5Eu sample. It shows a 3–4 times higher relative Eu concentration than that in the ZnO:Eu crystals. This indicates the higher Eu concentration at the grain-boundary was formed by expulsion of Eu-oxide from the highly Eu-doped metastable ZnO:Eu crystals. The 20Eu sample is expected from a structural point of view to be able to include at most 10–15% Eu as 4 to 8 Eu atom-sized clusters in the ZnO crystals as there would not be enough ZnO to cover the clusters otherwise. The XRD and IR data indicated that the sample had at least some material with a different doping mode besides that observed for the 5 and 10Eu sample. This difference is connected to the Eu-rich ZnO:Eu crystal boundary phase observed mainly in the 20Eu sample and also to some extent in 5Eu. The presence of this phase in the 5Eu sample may be due to the higher temperature used in this study compared to the previous one; 650 °C in the present study, compared to 600 °C in the previous study.^21,22^ It was previously shown by TEM that the first very rare 2–3 nm sized Eu_2_O_3_ crystals were formed at the ZnO:Eu sponge surface upon heating at higher temperature (700 °C).^21,22^ A more easy adaptation of the Eu-oxygen-coordination polyhedra and cluster is expected in the thin amorphous inter ZnO:Eu-crystal boundaries, not the least where ZnO:Eu crystals with misaligned lattice directions meet. For comparison, the 5–10 Å thickness in the 20Eu sample corresponds to ca. 2–4 Eu/ZnO_*x*_ units. The intercrystalline phase may also include ZnO.

**Figure 4:**
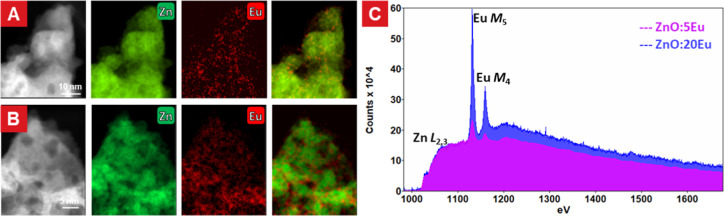
EELS-STEM analysis of 5Eu and 20Eu samples. (A,B) Elemental mapping of Zn L_2,3_ edge and Eu M_4,5_ edge of 5Eu (A) and 20Eu (B). (C) EELS spectra of the region of interest in 5Eu and 20Eu.

STEM-EELS was applied to explore the chemical properties of Eu. Figure 4A,B shows the elemental distributions of Zn and Eu. The Eu-rich region being much more pronounced for the 20Eu samples, as revealed with HAADF-STEM and EDX, was confirmed with the STEM-EELS mapping. The electron orbital occupancy can be calculated by analyzing the integrated intensity ratio between specific peaks in the EELS spectrum, thereby allowing for determination of the chemical valence states of selected elements and composition of the material. The signals in the EELS mapping were combined to get a high signal-noise ratio for chemical analysis. Figure 4C shows the summed EELS profiles of the 5 and 20Eu samples, respectively. Here, the EELS spectrum was normalized using the integral intensity of Zn (ranging from 1020 to 1121 eV) and compared with the relative intensity of the Eu signal. In concert with EDX data, it was found that the Eu concentration in the 5Eu sample was not as high as for the 20Eu sample. The peak shape and the strong white-line features in both spectra indicated that the europium was present as Eu-ions, rather than pure metal which should show weaker peaks around 1130 eV. From the literature, EuO and other Eu^2+^ compounds are unlikely to be present here as they are very susceptible to oxidation under the synthesis conditions used here.^30^ Furthermore, by comparing the relative intensity ratio between the Eu M_4_ and M_5_ edges, the valence state of europium could be set as +3, which is in accordance with the literature.^21,22,31^ Moreover, the determination of chemical state of Eu also helps for accurate calculation of atomic electron scattering factor.

### 3.3 Electron Pair-Distribution Function (ePDF) Analysis

**Figure 5:**
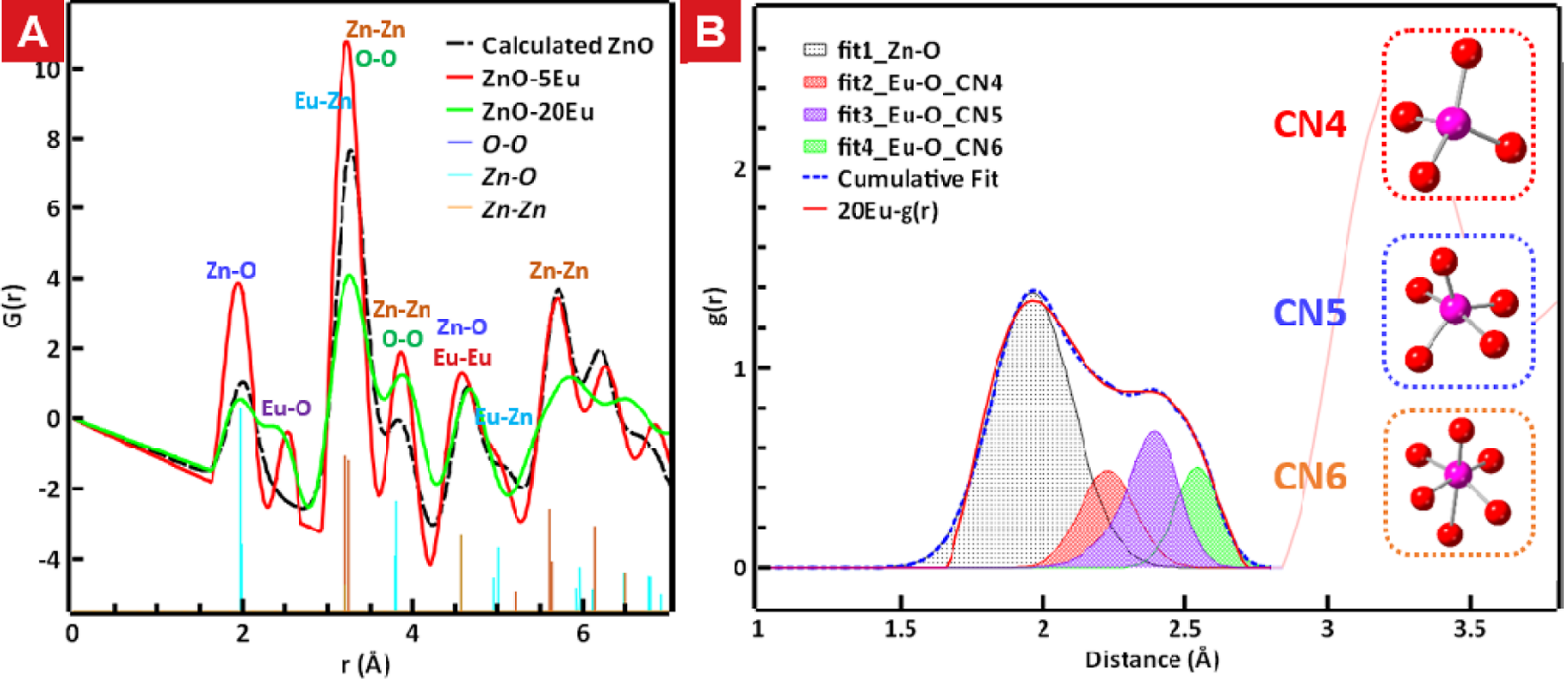
PDF profiles of the calculated ZnO and experimental 5Eu and 20Eu data. (A) Reduced-PDF profiles of calculated ZnO and experimental 5Eu and 20Eu. (B) Deconvolution of the peaks at the range of r between 2.1 and 2.8 Å in the 20Eu sample. The inset figures show the different Eu–O coordination configurations, corresponding to each deconvolution peak.

As discussed above, the distribution of Eu and Zn atoms in the 5Eu and 20Eu nanosponges was obtained from micrometer to nanometer scale by the HAADF-STEM and EELS studies. To get a closer insight into the local atomic structure, the interatomic distances were investigated with ePDF. Figure 5A shows the calculated ePDF of a standard *h*-ZnO structure (PDF card 00-036-1451) and the experimental ePDF of the 5Eu and 20Eu samples. No strong texture features were observed in diffraction patterns (Figures S9 and S10), which exclude the texture-lead inaccuracy of the relative intensity.

The most obvious differences between the 5Eu and 20 Eu samples and the *h*-ZnO rPDF were the peaks found at *r* = 2.1–2.8 Å, which were absent in the calculated *h*-ZnO rPDF. The peaks at longer distances showed significant variations between the samples but as these peaks originate from several different atom-pairs having similar distances, these peaks were more difficult to analyze in detail. Tentative assignments of the atom-pairs contributing to the composite peaks in the range *r* = 1.5–5.5 Å was made based on the DFT-generated structures with ZnO containing 4.1% Eu as Eu4 or Eu8 oxide clusters^22^ and a calculated *h*-ZnO structure as follows: 1.9–2.1 Å (Zn–O), 2.1–2.8 Å (Eu–O), 3.0–3.7 Å (Eu–Zn, Zn–Zn, O–O, Eu–Zn, Eu–O, Eu–Eu), 3.7–4.2 Å (Eu–O, Eu–Eu, Zn–O, Eu–O), 4.2–4.7 Å (Zn–Zn, O–O, Zn–O, Eu–Eu, Eu–Zn), 4.7–5.3 Å (Shoulder) (Eu–Zn, Zn–O, O–O), and 5.2–5.8 Å (Zn–Zn, O–O, Eu–Zn, Eu–Eu).

The ZnO:Eu samples and calculated *h*-ZnO *r*PDF showed a clear Zn–O peak at 1.97 Å (5Eu)–2.02 Å (20Eu), which was overlapped by the broad Eu–O peak in the latter case. This peak fits the Zn–O distances given as 1.974–1.989 Å for *h*-ZnO (PDF card: 00-036-1451) well. The Zn^2+^-ions of the *h*-ZnO are all tetrahedral four-coordinated by oxygen ions, and the Zn–O distances also match the sum of the ionic radii of coordination number 4 (CN4) Zn^2+^-and O^2–^ions (60 + 140 pm).^32^ A clear difference between the two ZnO:Eu samples could be observed in the Eu–O peaks. The 5Eu sample showed a single Eu–O peak at 2.2–2.7 Å, with a maximum at 2.51 Å. These Eu–O distances are in fair agreement with those obtained in the theoretical 4.1Eu structures containing oxide clusters with 4 or 8 Eu atoms.^22^ The Eu-oxide clusters contain Eu^3+^-ions coordinated mainly by five or six oxygen atoms, but in some cases, a four-coordinated Eu-ion is present. This led to very unusual low average Eu C.N. of 5.2–5.5 for the theoretic structures fitting the experimental data quite well and yielding the lowest energy.^22^ The 20Eu sample showed a much wider Eu–O peak covering distances from about 2.1 to 2.8 Å, with the peak shape at the longest distances of the band being similar to that of the 5Eu sample and a maximum at 2.40 Å. This broad band could tentatively be deconvoluted into serval peaks corresponding to different Eu–O coordination environments obtained from the modeled 4.1Eu structures (Figure 5B). The shortest Eu–O (Eu–O CN4) distances at ca. 2.1–2.4 Å fit the 2.22–2.26 Å found for four-coordinated Eu-ions in the theoretic Eu-oxide clusters,^22^ as well as the extrapolated sum of four-coordinated Eu-ions (0.84 Å)^21^ and oxygen-ions (1.40 Å)^32^ of 2.24 Å. The peak component at 2.3–2.5 Å was supplemented by a smaller component at 2.1–2.6 Å, to fit the ePDF of the 20Eu in this region. The CN5 peaks were fitted as asymmetry condition are in good agreement with 2.12–2.45 Å obtained for five-coordinated theoretic clusters in 4.1Eu structures, as well as the bond distance of 2.29 Å obtained from the extrapolated ionic radii of five-coordinated Eu^3+^-ions (0.89 Å) and O^2–^ ions (140 pm).^21^ The longest Eu–O bonds were in the range 2.4–2.6 Å, which is in line with the ca. 2.50 Å obtained from the six-coordinated Eu^3+^-ions in the theoretical 4.1Eu structures. This yields a Eu^3+^ ionic radius of 1.10 Å, which is longer than the reported experimentally obtained ionic radius of the Eu-ion (0.94 Å). The difference in ionic radii found for six-coordinated Eu-ions in the literature and the present and previously obtained experimental and theoretic structures may stem from the former being close packed crystalline structures while the latter are part of nonperiodic Eu-oxide clusters containing differently coordinated Eu-ions adapting their coordination sphere to fit the ZnO lattice which shows very little change due to the dopant clusters.

It showed that the 20Eu sample contains a broad range of Eu-ion coordination modes with a significantly increased fraction of Eu-ions in the very low four- and five-coordination to oxygen, compared to the 5Eu sample in which clusters were dominated by five- and six-coordinated Eu-ions, yielding typical average Eu C.N. of 5.2–5.5.^22^ However, it cannot be ruled out that there might also be higher coordinated Zn^2+^-ions present, which based on the reported ionic radii of Zn^2+^ (0.88 Å) and O^2–^ (1.40 Å)^32^ could have Zn–O bond distances of up to 2.28 Å for the six-coordinated Zn^2+^-ions. This distance would overlap with the shortest distances for the four-coordinated Eu^3+^-ions. Thus, the ePDF for the 5Eu sample gave results in good agreement with the previously obtained theoretical 4.1Eu structures with four or eight Eu atom oxide clusters occupying space in the *h*-ZnO crystal with nearly no effect on its unit cell-dimensions but introduced some short-range disorder with the range of 0–5 Å (Figure S8). Interestingly, it was found that the 20Eu sample, while being similar to the 5Eu sample for the longer Eu–O bond lengths, showed a substantial increase in Eu-ions coordinated with the very low four and five oxygen. However, the atom–atom distances of the four-coordinated Eu-ions overlap those of five- and six-coordinated Zn-ions, and therefore, it cannot be ruled out that the 20Eu sample also contained some higher Zn-ion coordination numbers than present in ZnO.

As the HAADF-STEM and EELS results present, this sample contained a substantial amount of an Eu-rich intercrystalline amorphous phase. The theoretical 4.1Eu structures reported imply that a maximum of 10–15% EuO_1.5_ clusters can be present in *h*-ZnO while providing enough ZnO to cover the clusters with one layer of Zn–O_4_ tetrahedral. The ca. 5–10 Å thick amorphous intercrystalline phase, to surround most ZnO:Eu crystals, could contain mainly low coordination Eu-ions and possibly some Zn-ions in higher five or six coordinations (CN5/6), besides possible four-coordinated Zn-ions. The latter CN4 Zn^2+^-ions would not be possible to distinguish those in the *h*-ZnO structure. The constraints of the ZnO crystal facets may affect the bonding between the crystalline and amorphous phases, and the various ionic radii of the metal ions involved are likely best satisfied by an amorphous Zn–Eu-oxide structure. The Zn^2+^- and Eu^3+^-ions having spherical d^10^ and s^2^ outer orbital shells without requirements of bond directions may make mixed coordination numbers and wide bond-length distributions facilitated. The different typical ionic sizes may be equalized in an amorphous structure by attaining the very low CN4 for Eu^3+^-ions (84 pm) and CN 5/6 for Zn^2+^-ions (74 pm/88 pm), which can allow for a tighter mixing of the ions. It can be mentioned that when looking at DFT-generated Eu2 clusters in ZnO containing four- and five-coordinated Eu-ions, the mismatch in size of the Eu-ions and the surrounding crystalline ZnO structure caused a much larger increase in ZnO:Eu unit cell-dimensions associated with an increase in energy. This was compared to the larger Eu-oxide clusters, which more easily could adjust coordination within the larger cluster occupied cavity and thereby give very low increase in ZnO:Eu unit cell parameters and result in the lowest lattice distortion. In the amorphous structure described above, there would not necessarily be a strain between the single Eu-ions or oxide clusters and ZnO as with the crystalline ZnO, and therefore, the formation energy may be lower compared to the crystalline case.

Nevertheless, the PDF [*g*(*r*)] were also obtained based on applying proper average number density (ρ_0_) (Figure S11). The calculated number densities for 5Eu and 20Eu were approximately 0.0728 and 0.0875 Å^–3^, respectively, which are comparable to the ideal number density of h-ZnO (0.0839 Å^–3^). Hence, the coordination number can be calculated and compared with EXAFS data from previous work^22^ (Table S2). However, small differences were observed, even though no strong texture features were shown in diffraction patterns (Figures S9 and S10). In this context, the ePDF provided adequately described structural characteristics of europium in nanosponges and exhibited a high precision in the average number density. However, the full PDF results diverge slightly from the experimental data obtained through EXAFS results in the literature.

## 4 Conclusions

In this work, the structural features in ZnO:Eu nanosponges were investigated from micrometer to atomic scale using electron microscope imaging, spectroscopy, and ePDF, as well as XRD and IR spectroscopy. It was found that at least 10% EuO_1.5_ could be introduced into *h*-ZnO with virtually no change in the overall structure compared to the 5Eu structure judging from the IR and XRD patterns, while addition of 20% EuO_1.5_ led to significant formation of an amorphous, 5–10 Å thick Eu-rich intercrystalline phase, besides the Eu-oxide dispersed as clusters in the ZnO:Eu crystals similar to the 5% EuO_1.5_ sample studied. The intercrystalline phase was found by HAADF, EDX, and EELS mapping, and the latter technique showed that Eu was trivalent in similarity with the Eu^3+^ in the ZnO:Eu crystals, which is in line with previous studies on 5% Eu samples.

The ePDF was applied to get quantitative information about structural deviations on Eu-doping in different samples. The PDF peak intensities provided Eu–O and Eu–Eu distances which for the higher doped 20Eu sample showed peaks at 2.0 and 3.2 Å, which is lower than that of 5Eu and indicates low concentration of Zn–O and Zn–Zn atom pair. The ePDF results are compatible well with all other experimental results above and fit the findings from previous studies. Moreover, the ePDF not only provided information on how structure deviated from a standard sample, as was acquired from STEM, but also gave precise atom–atom bonding information. The combination of imaging, spectroscopy, and ePDF offers a unique, in-depth spatial understanding of structural variations caused by different doping levels. This powerful set of techniques enables detailed material characterization, including both crystalline and amorphous phases in complex compositions, at sizes as small as a nanometer—providing insights beyond the capabilities of standard techniques.

## Data Availability

A version of this manuscript was submitted to *ChemRxiv*: 10.26434/chemrxiv-2024-dmpvq.^33^

## Supporting Information Available

Detailed XRD unit cell information on ZnO-Eu, EDX and related spectra, ED patterns, and scattering profiles of ZnO-Eu nanosponges (PDF)
