# Supplementary material for: Atomistic Structure Investigation of Eu-Doped ZnO Nanosponges
Source: Inorg Chem. 2025 Jan 2;64(1):232–41. doi: 10.1021/acs.inorgchem.4c04494 (PMC11734123; doi:10.1021/acs.inorgchem.4c04494)
Supplement: Supplementary file 1 — ic4c04494_si_001.pdf [file ic4c04494_si_001.pdf]

Supporting Information

Atomistic structure investigation of Eu-doped ZnO nano-sponges

Shihui Feng,[1]* Sarmad Naim Katea ,[2] Markus Ek,[2] Gunnar Westin,[2] Cheuk-Wai Tai[1]*

[1]Department of Material and Environmental Chemistry, Stockholm University, Svante Arrhenius vag 16C, Stockholm, SE106-91, Sweden
[2]Department of Chemistry-Ångström, Ångström Laboratory, Uppsala University, Uppsala, SE751-21, Sweden

* corresponding authors: shihui.feng@mmk.su.se and cheuk-wai.tai@mmk.su.se

Table S1. Unit cell-parameters for *h*-ZnO:Eu.

ZnO:Eu (%Eu)	0	5	10	20
a (Å) 600 °C	3.25076 (8)	3.2518 (3)	3.2494 (6)	3.246 (1)
a (Å) 650 °C	3.2478 (1)	3.2515 (3)	3.2497 (6)	3.247 (1)
c (Å) 600 °C	5.2081 (1)	5.2054 (5)	5.202 (1)	5.196 (2)
c (Å) 650 °C	5.2027 (3)	5.2040 (4)	5.201 (1)	5.199 (2)
Vol. ($Å^3$) 600 °C	47.662 (3)	47.670 (1)	47.57 (2)	47.41 (4)
Vol. ($Å^3$) 650 °C	47.528 (4)	47.650 (1)	47.57 (2)	47.48 (4)

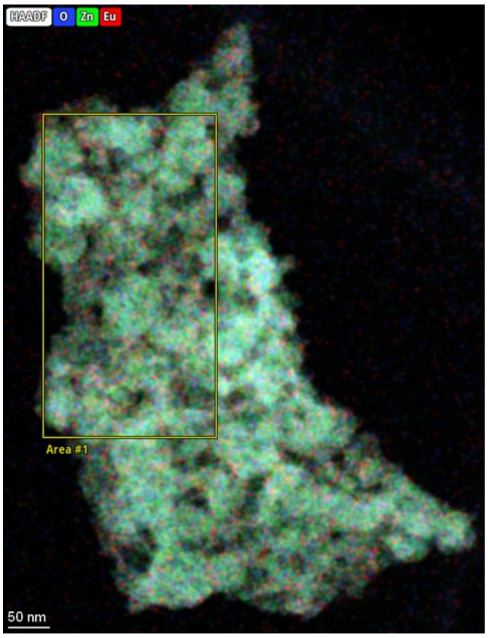

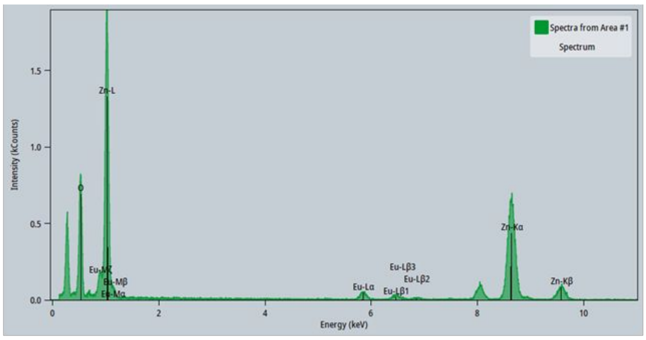

Element	Atomic Fraction (%)	Atomic Error (%)	Mass Fraction (%)	Mass Error (%)
O	57.36	3.12	23.66	2.18
Zn	40.67	3.22	68.59	2.93
Eu	1.98	0.24	7.74	1.05

Figure. S1. Elemental mapping of ZnO: 5Eu nanosponges. The EDX spectrum and normalized element composition of the highlight region are shown. The unidentified peak around 8 keV is the Cu K_{α} signal generated by the TEM grid.

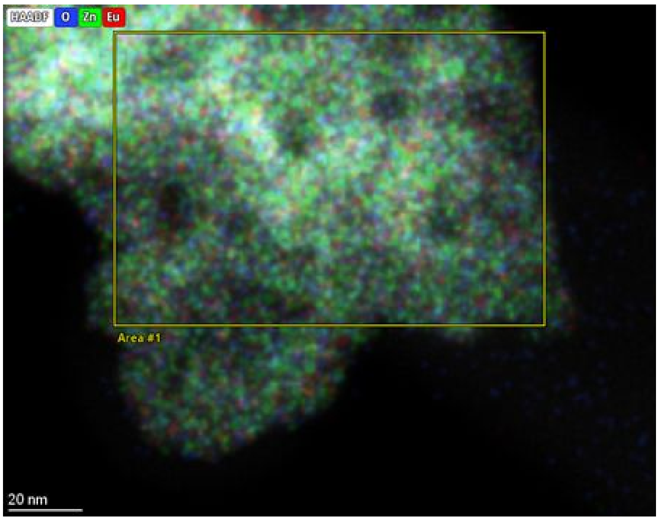

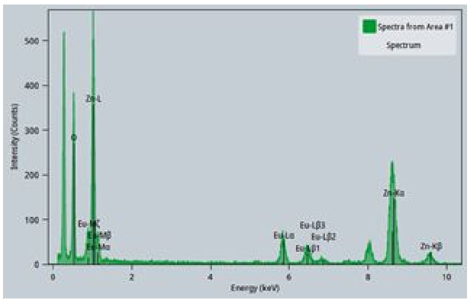

Element	Atomic Fraction (%)	Atomic Error (%)	Mass Fraction (%)	Mass Error (%)
O	57.71	2.88	21.90	1.78
Zn	36.22	3.14	56.20	3.56
Eu	6.07	0.69	21.90	2.49

Figure. S2. Elemental mapping of ZnO: 20Eu nanosponges. The EDX spectrum and normalized element composition of the highlight region are shown. The unidentified peak around 8 keV is the Cu K_{α} signal generated by the TEM grid.

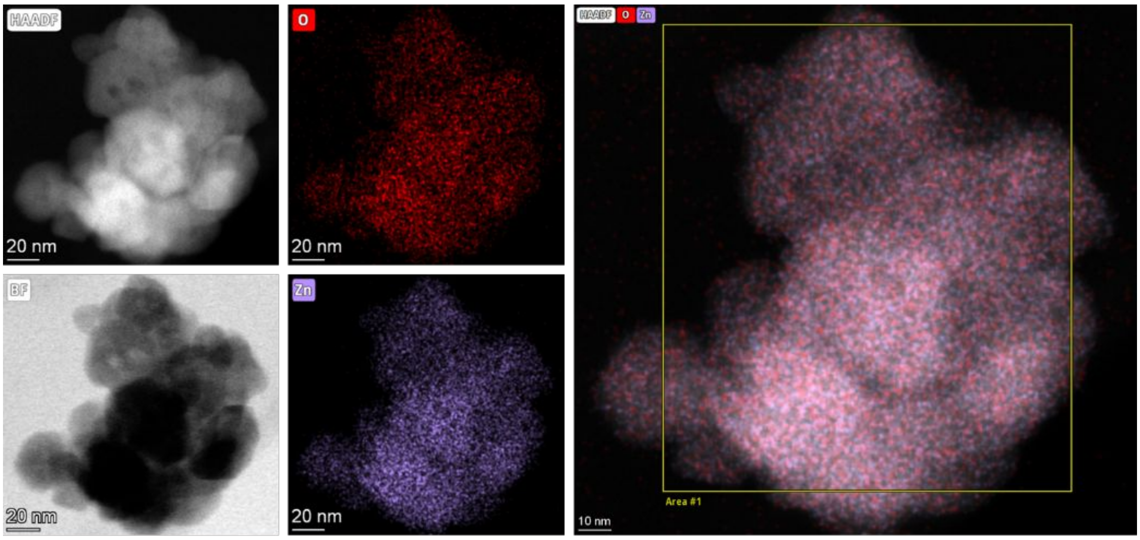

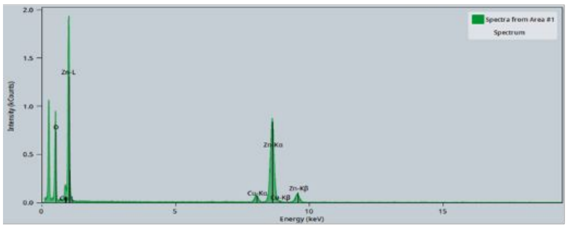

Element	Atomic Fraction (%)	Atomic Error (%)	Mass Fraction (%)	Mass Error (%)
O	53.56	3.37	22.00	2.32
Zn	46.44	3.37	78.00	2.32

Figure. S3. Elemental mapping of ZnO nanosponges. The EDX spectrum and normalized element composition of the highlight region are shown.

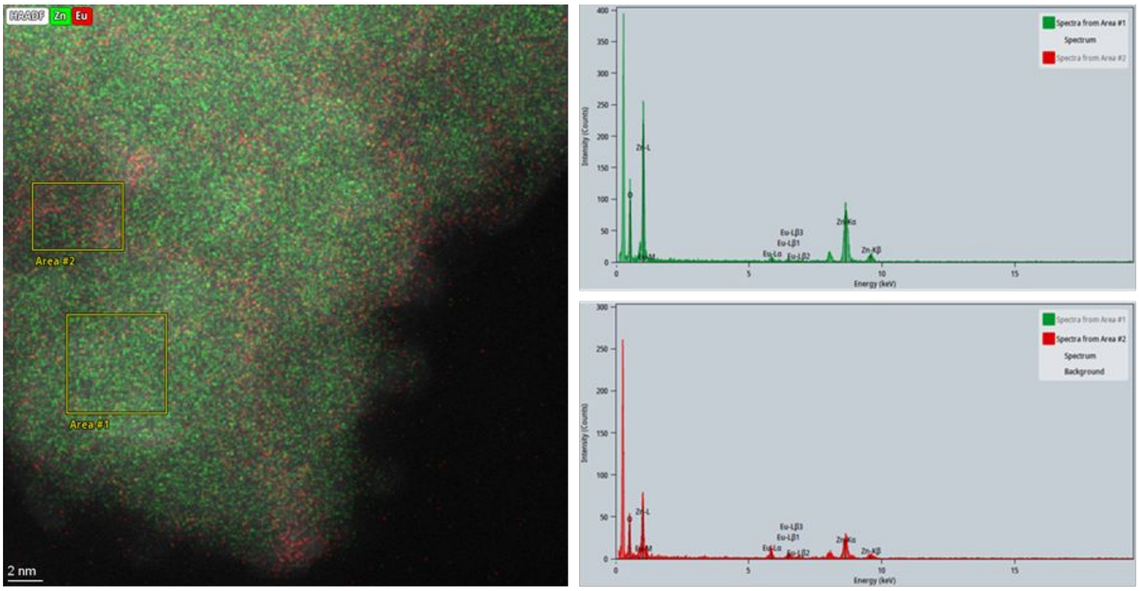

Area 1	Atomic Fraction (%)	Atomic Error (%)	Mass Fraction (%)	Mass Error (%)
O	60.37	3.11	26.19	2.40
Zn	38.12	3.18	67.60	2.98
Eu	1.51	0.18	6.21	0.86

Area 2	Atomic Fraction (%)	Atomic Error (%)	Mass Fraction (%)	Mass Error (%)
O	65.09	2.61	27.16	1.98
Zn	29.03	2.79	49.52	3.59
Eu	5.88	0.66	23.32	2.51

Figure. S4. Supplementary EDX spectra and corresponding element composition of ZnO:5Eu nanosponges in Figure 5B. EDX spectra were obtained from the highlighted regions in the overlay. The table below shows the relative element composition of the selected regions in the mapping.

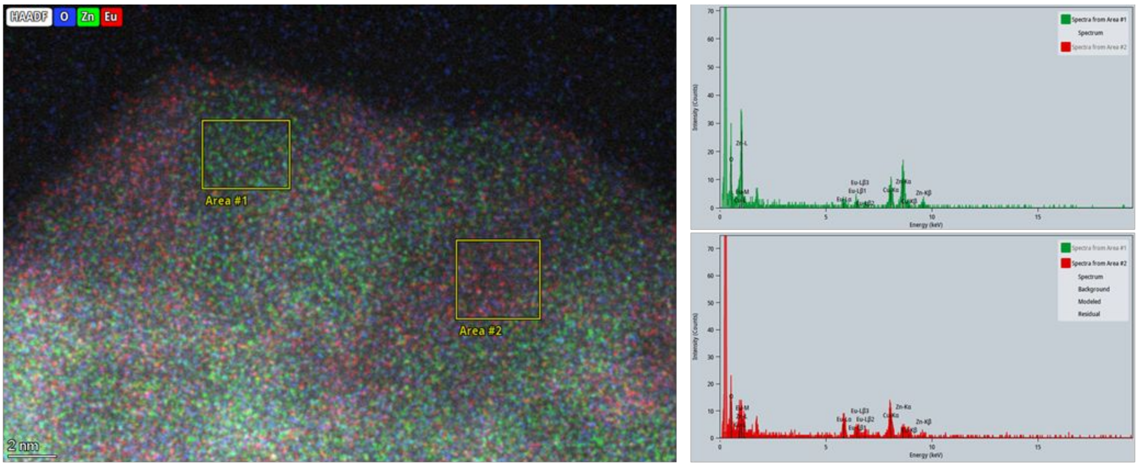

Area 1	Atomic Fraction (%)	Atomic Error (%)	Mass Fraction (%)	Mass Error (%)
O	66.17	2.80	29.71	2.37
Zn	30.46	2.91	55.91	3.51
Eu	3.37	0.44	14.38	1.89

Area 2	Atomic Fraction (%)	Atomic Error (%)	Mass Fraction (%)	Mass Error (%)
O	76.49	1.65	31.53	2.02
Zn	10.58	1.37	17.83	2.32
Eu	12.93	1.29	50.63	3.09

Figure. S5. Supplementary EDX spectra and corresponding element composition of ZnO:20Eu nanosponges in Figure 5G. EDX spectra were obtained from the highlighted regions in the overlay. The table below shows the relative element composition of the selected regions in the mapping.

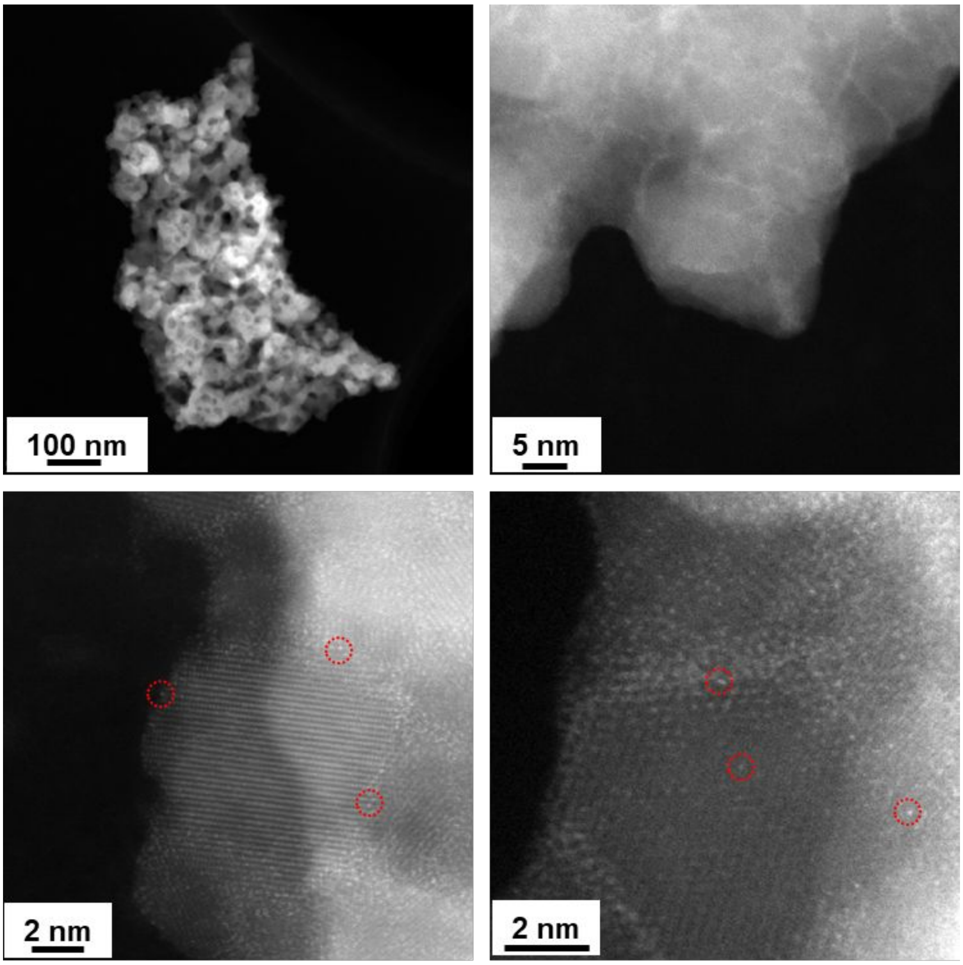

Figure. S6. Additional STEM data of ZnO:5Eu sample. The Eu atoms were marked by red circles.

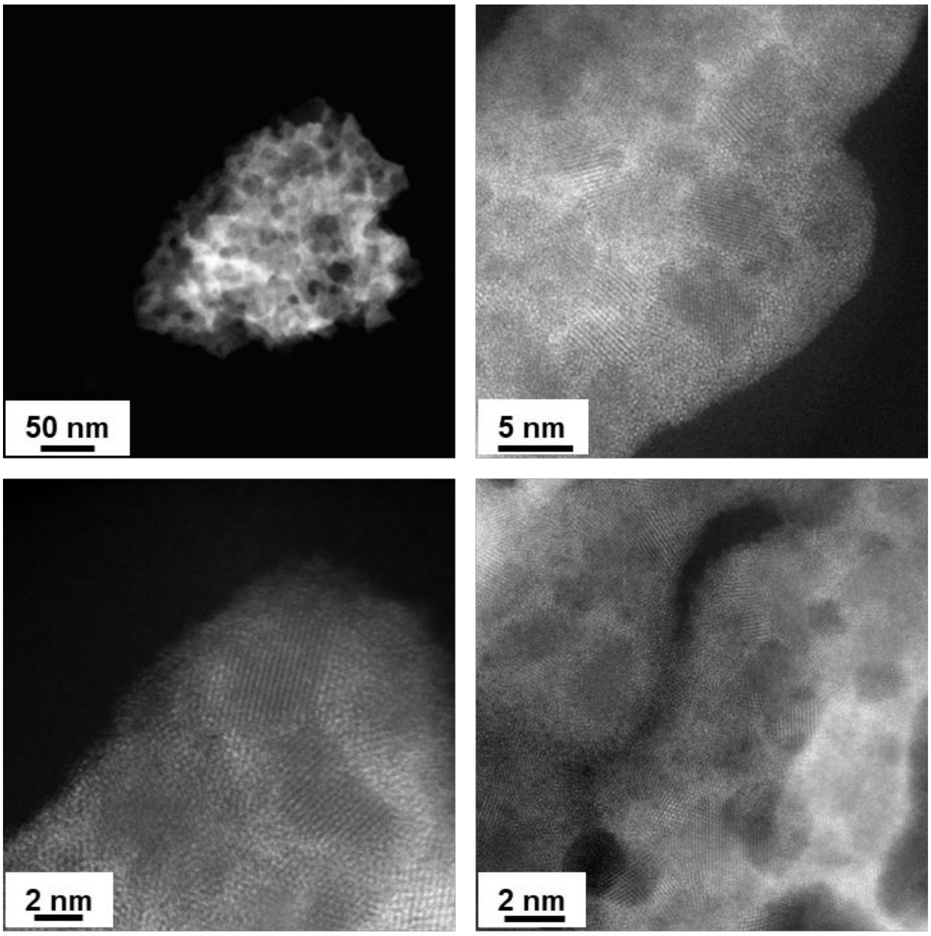

Figure. S7. Additional HAADF-STEM images of ZnO:20Eu sample.

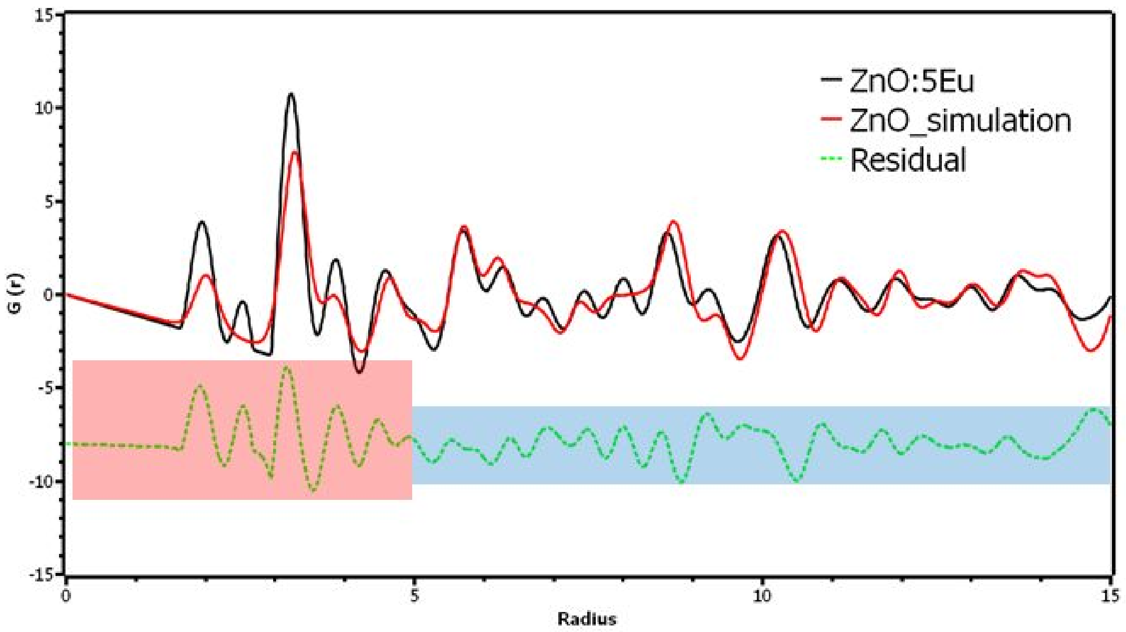

Figure. S8. Comparison of the experimental and theoretical reduced PDF. The differences are presented as the green dash line. The radius range of 0-5 Å and of 5 -15 Å, marked in red and blue respectively, indicates the short-range and medium-range order region, respectively.

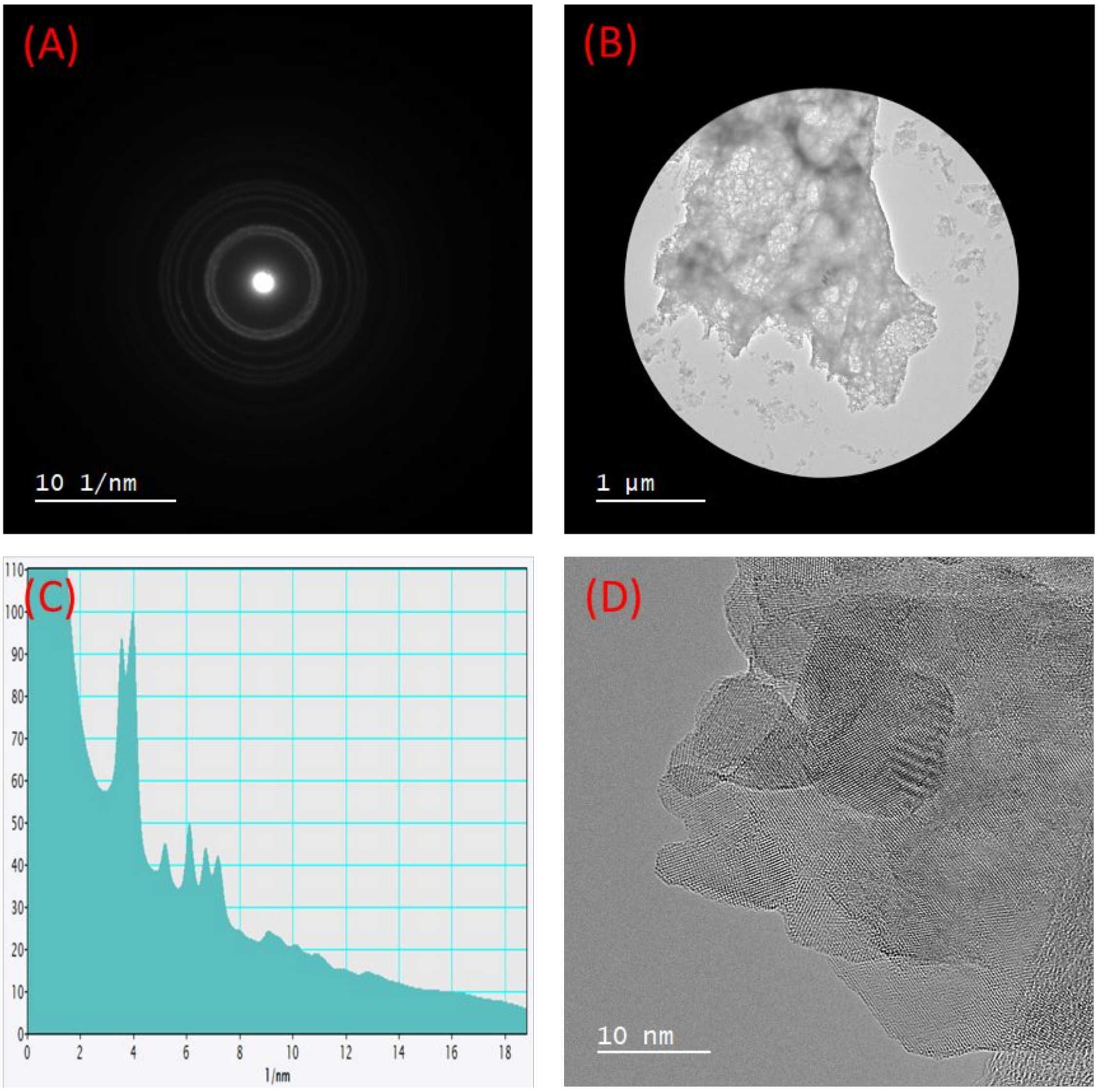

Figure. S9. (A) SAED pattern of the region of interested (B) in ZnO-5Eu. (C) Azimuthal integration of the SAED pattern was used for ePDF analysis. (D) Representative HRTEM image of ZnO:5Eu

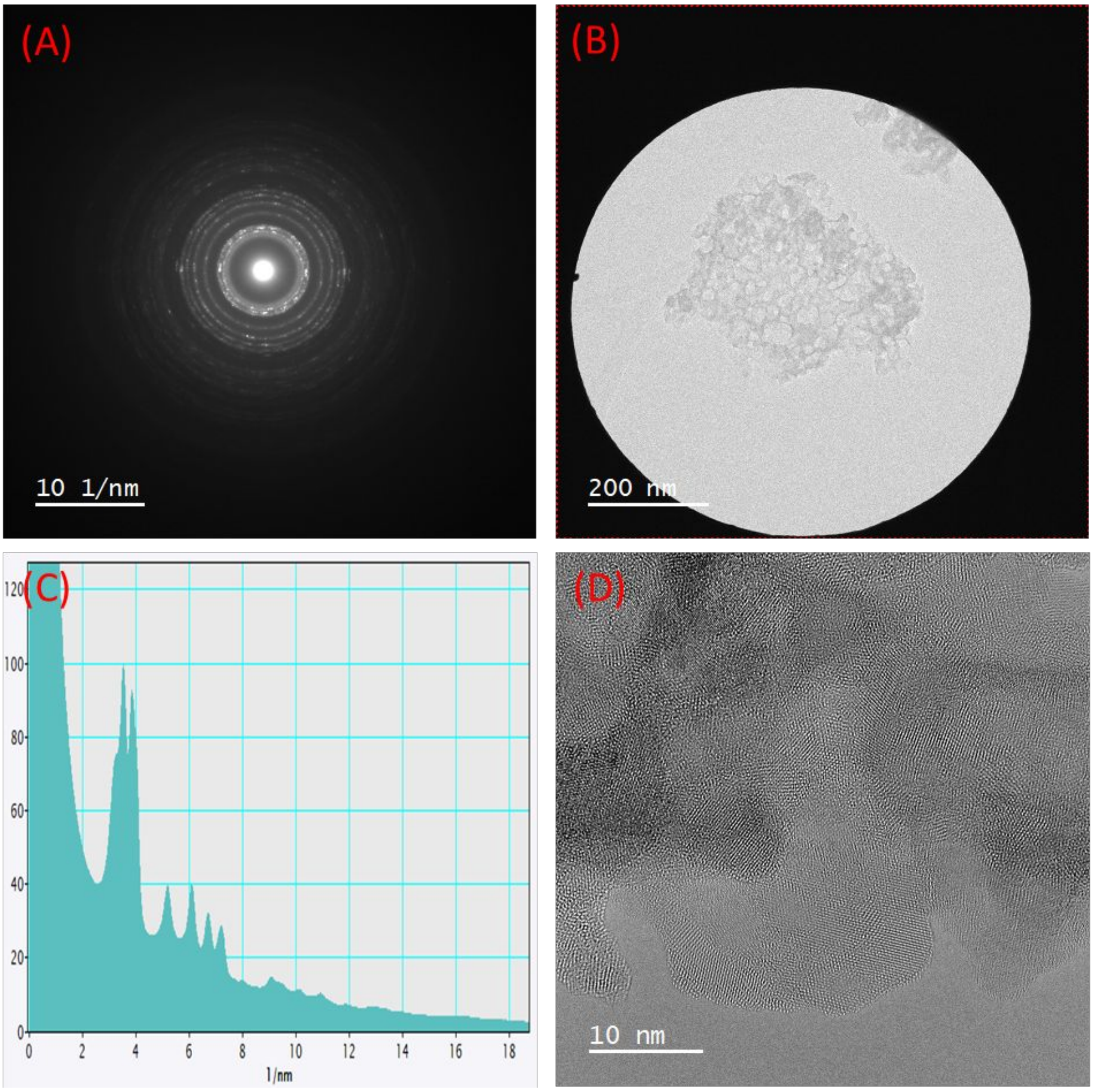

Figure. S10. (A) SAED pattern of the region of interested (B) ZnO-20Eu. (C) Azimuthal integration of SAED pattern. (D) Representative HRTEM image of ZnO:20Eu

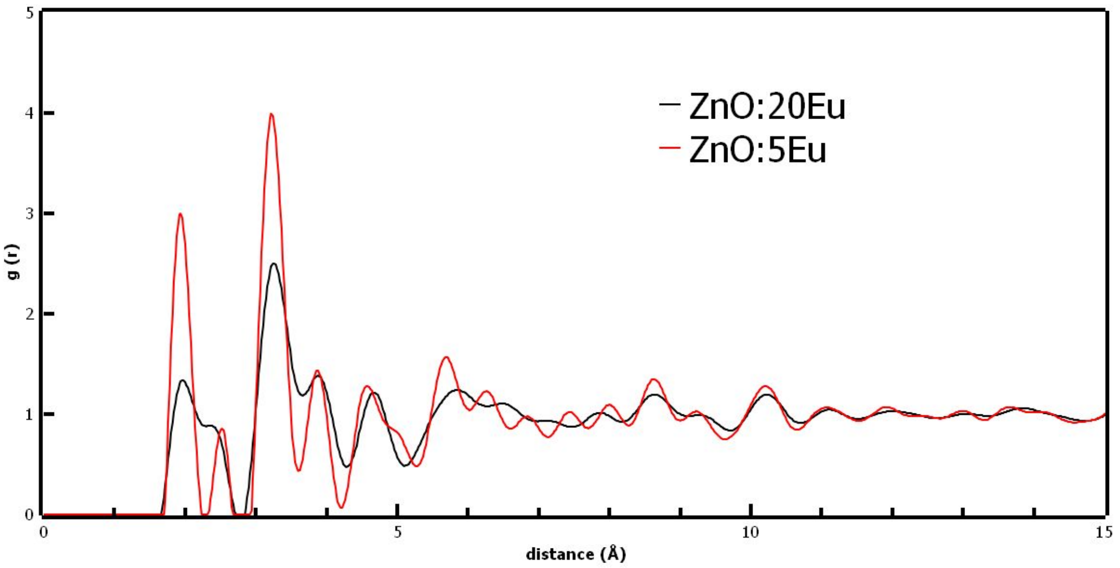

Figure. S11. Full PDF profile *g(r)* of 5Eu and 20Eu nanosponges, the calculated number density for 5Eu and 20Eu were 0.915± 5.5×10^{-7} and 1.11± 7.6×10^{-7} $Å^{-3}$, respectively.

Table S2. Calculated coordination number of different atom pairs

	Zn-O (2.005 Å)	Eu-Eu/Eu-O (2.2 -2.5 Å)
5Eu	5.56	3.41
20Eu	2.88	2.26
Literature	4.0*	5.2

* The 1st coordination shell (Zn-O) was not studied in the literature. Consider no Eu-O, Zn-Eu and Eu-Eu distance were below 2.0 Å, so the standard coordination value from ideal hexagonal-ZnO model (4.0) was used in this case.
